# Analysis of Epinephrine Dose, Targeted Temperature Management, and Neurologic and Survival Outcomes Among Adults With Out-of-Hospital Cardiac Arrest

**DOI:** 10.1001/jamanetworkopen.2022.26191

**Published:** 2022-08-11

**Authors:** Betty Y. Yang, Natalie Bulger, Richard Chocron, Catherine R. Counts, Chris Drucker, Lihua Yin, Megin Parayil, Nicholas J. Johnson, Nona Sotoodehenia, Peter J. Kudenchuk, Michael R. Sayre, Thomas D. Rea

**Affiliations:** 1 Department of Emergency Medicine, University of Washington, Seattle; 2 Department of Emergency Medicine, Georges Pompidou European Hospital, Assistance Publique-Hôpitaux de Paris, Paris Sudden Death Expertise Center, University of Paris, Paris, France; 3 Emergency Medical Services, Division of Public Health–Seattle & King County, Seattle, Washington; 4 Division of Pulmonary, Critical Care, and Sleep Medicine, Harborview Medical Center, University of Washington, Seattle; 5 Division of Cardiology, Department of Medicine, University of Washington, Seattle; 6 Division of General Medicine, Department of Medicine, University of Washington, Seattle

## Abstract

**Importance:**

Epinephrine improves return of spontaneous circulation after out-of-hospital cardiac arrest (OHCA). These beneficial cardiac effects do not directly translate to better neurologic outcomes, possibly because of epinephrine-induced microvascular effects that produce critical brain ischemia.

**Objective:**

To examine whether targeted temperature management (TTM) modifies the adverse association between increasing prehospital epinephrine dose and neurologically favorable survival.

**Design, Setting, and Participants:**

This retrospective cohort study assessed 14 612 adults from Seattle and King County, Washington, with nontraumatic OHCA between January 1, 2008, and December 31, 2018, and included those who achieved return of spontaneous circulation and were unconscious at hospital admission. Data analysis was performed from April 2021 to May 2022.

**Exposures:**

Epinephrine dose and TTM during prehospital resuscitation.

**Main Outcomes and Measures:**

Favorable neurologic survival (Cerebral Performance Category [CPC] 1 or 2) and survival to hospital discharge.

**Results:**

Of the 14 612 assessed adults, 5253 (median age, 63 years; IQR, 51-74 years; 3460 [65.8%] male) were eligible for the study. The median epinephrine dose was 2.0 mg (IQR, 1.0-3.0 mg); 3052 patients (58.1%) received TTM. In all, 1889 patients (36.0%) survived with CPC 1 to 2, and 2177 (41.4%) survived to discharge. Increasing doses of epinephrine were associated with a decreasing likelihood of CPC 1 to 2 (odds ratio [OR], 0.46; 95% CI 0.42-0.50 for each additional milligram of epinephrine) and survival (OR, 0.47; 95% CI, 0.43-0.51). The dose-dependent epinephrine association was modified by TTM. After adjusting for Utstein covariates, TTM was associated with a relative stepwise improvement in odds of CPC 1 to 2 (interaction OR, 1.36; 95% CI, 1.22-1.51) and survival (interaction OR, 1.37; 95% CI, 1.24-1.51). A significant interaction was also observed when the analysis was stratified according to initial rhythm among shockable OHCA and nonshockable OHCA (shockable interaction OR, 1.20; 95% CI, 1.04-1.39; and nonshockable interaction OR, 1.24, 95% CI, 1.07-1.45).

**Conclusions and Relevance:**

This cohort study found an interaction between TTM and epinephrine dose such that the beneficial association of TTM increased with increasing epinephrine dose, suggesting that TTM may attenuate the adverse effects of higher-dose epinephrine.

## Key Points

**Question:**

Does targeted temperature management (TTM) modify the association between higher epinephrine doses and poor survival outcomes after out-of-hospital cardiac arrest?

**Findings:**

This cohort study of 5253 adults with out-of-hospital cardiac arrest found a statistically significant interaction between TTM and increasing epinephrine dose on neurologically favorable survival, such that the relative beneficial association of TTM increased with each additional 1-mg dose of epinephrine during prehospital resuscitation.

**Meaning:**

Targeted temperature management may attenuate the adverse outcome associated with higher-dose epinephrine and help better direct therapy to improve survival.

## Teaser

This cohort study of adults with out-of-hospital cardiac arrest examines whether targeted temperature management modifies the association between increasing prehospital epinephrine dose and poor neurologic and survival outcomes.

## Introduction

Out-of-hospital cardiac arrest (OHCA) is a major cause of mortality worldwide.^1,2^ Successful resuscitation and survival are possible and rely on a timely coordinated response that involves early recognition, early cardiopulmonary resuscitation (CPR), early defibrillation, and both expert advanced life support (ALS) and meticulous postresuscitation care.^1,3^

A mainstay of ALS has been the use of epinephrine to help restore spontaneous circulation. In a large randomized clinical trial^4^ of epinephrine vs placebo for OHCA resuscitation, epinephrine substantially increased return of spontaneous circulation (30% vs 12%) but produced a modest survival-to-discharge benefit (3% vs 2%) and no effect on neurologically favorable survival (2% vs 1.5%). One potential explanation is that epinephrine’s cardiac benefits are attenuated by its adverse neurologic effects. More specifically, epinephrine may not only improve macrovascular coronary flow, cardiac electrical activity, and myocardial contractility but may also induce microvascular dysregulation that worsens critical brain ischemia.^5,6^ This balance of benefit and risk may become increasingly unfavorable as the total dose of epinephrine increases, although timing of administration may also influence outcome.^7,8^

Targeted temperature management (TTM) may support brain recovery after out-of-hospital cardiac arrest (OHCA), presumably by limiting the cascade of neuronal damage and death activated by the ischemia of the arrest and potentially worsened by reperfusion injury, postresuscitation systemic inflammation, and, potentially, increasing dose of epinephrine.^9,10^ Although previous trials^11,12,13,14,15^ have demonstrated conflicting results regarding the optimal temperature, data from 2 large retrospective studies^14,15^ suggest that the benefit of TTM might be greatest in patients with more profound ischemia-reperfusion injury.

Whether TTM can mitigate the adverse neurologic effects associated with epinephrine is uncertain. We hypothesized that an interaction occurs between epinephrine dose and TTM such that the adverse association between increasing epinephrine dose and neurologically favorable survival is attenuated by TTM or, conversely, the potential survival benefit of TTM increases as the dose of epinephrine increases.

## Methods

### Study Design, Population, and Setting

**Figure 1.**
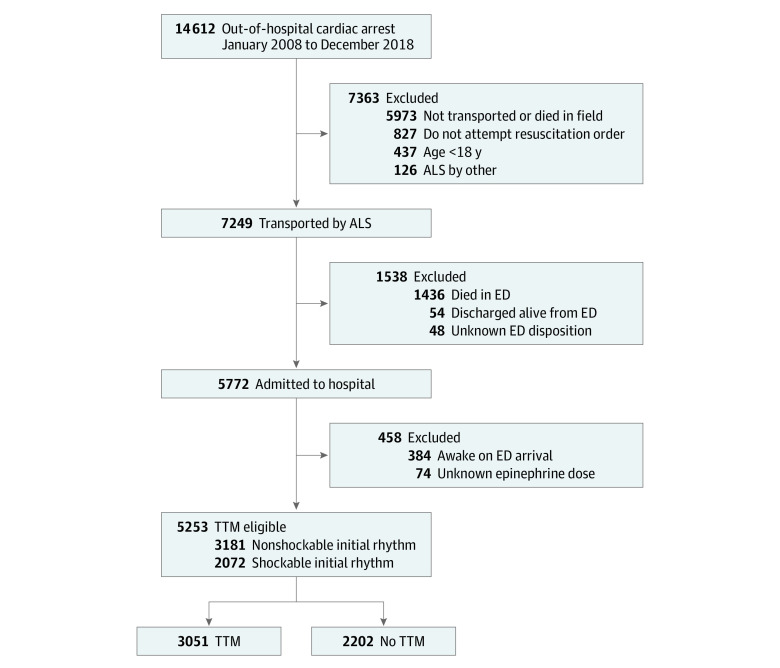
Flow Diagram of Patient Cohort ALS indicates advanced life support; ED, emergency department; and TTM, targeted temperature management.

This retrospective cohort study assessed 14 612 adults (≥18 years of age) with nontraumatic OHCA who were potentially eligible for TTM in Seattle and King County, Washington, from January 1, 2008, to December 31, 2018 (Figure 1). To be eligible for TTM, patients had to be admitted to the hospital after resuscitation and not awake on emergency department (ED) arrival. Patients who were not eligible for TTM were excluded, including patients who were pronounced dead in the field or ED or who were discharged alive from the ED. Other exclusions included those who received ALS care by agencies outside King County, those who had a do-not-attempt-resuscitation order, and those for whom epinephrine dose was unknown. The study was reviewed and approved by the University of Washington Institutional Review Board and by the Research Review Committee of Public Health–Seattle & King County. Because the investigation was observational and considered minimal risk, the requirement for consent was waived. This study was conducted in accordance with the Strengthening the Reporting of Observational Studies in Epidemiology (STROBE) guideline.^17^

King County is a large metropolitan region that covers 2300 square miles, with 2.3 million persons who reside in urban, suburban, and rural areas. Each year, approximately 1300 persons receive attempted resuscitation from emergency medical services (EMS) after OHCA. Resuscitation generally follows the American Heart Association guidelines.^16^ First-tier emergency medical technicians provide CPR and use automated external defibrillators (AEDs). Second-tier paramedics are trained in advanced cardiac life support, including manual rhythm assessment, advanced airway placement, and administration of advanced cardiac life support medications, such as epinephrine. Patients are transported to 1 of 14 hospitals equipped with coronary intervention and critical care capabilities, including TTM.

### Data Collection and Definitions

Emergency medical services maintains a registry of every treated OHCA organized according to the Utstein template.^18^ Trained coordinators review dispatch recordings, EMS records, defibrillator recordings, and hospital information to code demographic, circumstance, care, and outcome information. Because we did not have a reliably accurate data source for race and ethnicity categories, we did not collect this data on this demographic characteristic for this study. Data are prospectively collected comprehensively to mitigate potential selection bias. Arrest duration was estimated based on the interval from the 9-1-1 call to initial return of spontaneous circulation for patients who arrested before EMS arrival and from scene arrival to initial return of spontaneous circulation for patients who arrested after EMS arrival.

### Exposures

The primary exposures were the total prehospital epinephrine dose and TTM. Epinephrine dose was determined from review of the EMS record, and TTM was defined as application of surface cooling pads or an endovascular cooling catheter based on review of the hospital record. Prehospital cooling was not performed during the study period.

### Outcome

The primary outcome was neurologically favorable survival at hospital discharge as assessed by the Cerebral Performance Category (CPC). The CPC was measured through review of the hospital records. We classified a CPC score of 1 or 2 as neurologically favorable survival. The CPC has good interrater reliability^19^ and is associated with long-term prognosis.^20^ The secondary outcome was survival to hospital discharge.

### Statistical Analysis

Standard statistical software (R, version 4.0.3, R Foundation for Statistical Computing) was used for all statistical analysis.^21^ Descriptive statistics were used to assess baseline characteristics overall and according to epinephrine dose and TTM status. Using multivariable logistic regression, we modeled the association of outcome, epinephrine dose, TTM, and interaction between epinephrine dose and TTM on outcome. Epinephrine dose was modeled as a continuous variable to optimize power. Secondary analyses also modeled epinephrine dose as a discrete independent categorical variable. We also performed a post hoc analysis to evaluate for a potential threshold effect of the epinephrine dose, which classified dose groups according to those who received 0 mg, more than 0 to 3 mg, and more than 3 mg.

Multivariable models adjusted for Utstein covariates, including age, sex, witness status (witnessed by EMS, witnessed by bystander, and unwitnessed), bystander CPR, AED application, initial rhythm (shockable vs nonshockable), and the interval from receipt of 9-1-1 call to initial responder scene arrival. To account for potential variation in care or outcome across hospitals, we used a hierarchical mixed-effects model with hospital modeled as a random effect. In a preplanned subgroup analysis, we stratified results according to initial rhythm: shockable vs nonshockable. In sensitivity analyses, we additionally adjusted for duration of arrest. Data analysis was performed from April 2021 to May 2022.

## Results

Of the 14 612 assessed adults, 5253 (median age, 63 years [IQR, 51-74 years; 3460 [65.8%] men and 1793 [34.2%] women) were included in the study. Those receiving TTM were more likely to be male, arrest before EMS arrival and in a public location, and present with a shockable rhythm (eTable 1 in the Supplement). The median epinephrine dose was 2.0 mg (IQR, 1.0-3.0 mg). A total of 3052 (58.1%) received TTM. When characteristics were stratified by epinephrine dose and TTM status (Table), the proportion who received TTM was smallest among the 0-mg epinephrine dose group (574 [48.8%]). Across the other epinephrine groups, the proportion of patients receiving TTM ranged from 58.0% (n = 450) for more than 2 to 3 mg to 66.1% (n = 283) for more than 3 to 4 mg.

**Table.** Patient Characteristics Stratified by Dose of Epinephrine and TTM^a^

Characteristic	Overall (N = 5253)	Epinephrine dose, mg											
		0		>0-1		>1-2		>2-3		>3-4		>4	
		TTM (n = 574)	No TTM (n = 603)	TTM (n = 751)	No TTM (n = 518)	TTM (n = 685)	No TTM (n = 403)	TTM (n = 450)	No TTM (n = 325)	TTM (n = 283)	No TTM (n = 145)	TTM (n = 308)	No TTM (n = 208)
Age, median (IQR), y	63 (51-74)	62 (50-71)	62 (50-75)	61 (51-73)	64 (52-75)	62 (51-74)	67 (55-80)	61 (50-73)	65 (53-77)	65 (56-75)	64 (54-77)	61 (51-70)	61 (50-73)
Sex													
Female	1793 (34.2)	161 (28.0)	246 (40.8)	271 (36.1)	216 (41.7)	210 (30.7)	163 (40.4)	127 (28.2)	122 (37.5)	86 (30.4)	53 (36.6)	73 (23.7)	65 (31.2)
Male	3460 (65.9)	413 (72.0)	357 (59.2)	480 (63.9)	302 (58.3)	475 (69.3)	240 (59.6)	323 (71.8)	203 (62.5)	197 (69.6)	92 (63.4)	235 (76.3)	143 (68.8)
Cardiac arrest	3480 (66.2)	477 (83.1)	331 (54.9)	539 (71.8)	254 (49.0)	503 (73.4)	215 (53.3)	336 (74.8)	164 (50.5)	221 (78.1)	84 (57.9)	237 (76.9)	118 (56.7)
Arrest before EMS arrival	4543 (86.5)	522 (90.9)	483 (80.1)	684 (91.1)	378 (73.0)	628 (91.7)	323 (80.1)	419 (93.1)	272 (83.7)	253 (89.4)	122 (84.1)	282 (91.6)	177 (85.1)
Public location	1356 (25.8)	220 (38.3)	175 (29.0)	225 (30.0)	103 (19.9)	186 (27.2)	66 (16.4)	117 (26.0)	56 (17.2)	68 (24.0)	28 (19.3)	75 (24.4)	37 (17.8)
Witnessed arrest^b^	2791 (61.4)	388 (74.3)	326 (67.5)	425 (62.1)	247 (65.3)	360 (57.3)	178 (55.1)	240 (57.3)	149 (54.8)	142 (56.1)	70 (57.4)	166 (58.9)	100 (56.5)
Bystander CPR^b^	3093 (68.1)	382 (73.2)	329 (68.1)	450 (65.8)	262 (69.3)	420 (66.9)	210 (65.0)	266 (63.5)	190 (69.9)	178 (70.4)	80 (65.6)	202 (71.6)	124 (70.1)
Shockable initial rhythm	2072 (39.4)	426 (74.2)	229 (38.0)	339 (45.1)	118 (22.8)	292 (42.6)	70 (17.4)	183 (40.7)	55 (16.9)	132 (46.6)	37 (25.5)	134 (43.5)	57 (27.4)
AED application	385 (7.3)	80 (13.9)	58 (9.6)	48 (6.4)	32 (6.2)	47 (6.9)	18 (4.5)	33 (7.3)	18 (5.5)	14 (4.9)	7 (4.8)	23 (7.5)	7 (3.4)
Call to EMS arrival, median (IQR), min	5.0 (4.0-6.4)	4.7 (3.9-6.0)	5.0 (4.0-6.3)	4.9 (4.0-6.1)	5.2 (4.0-6.9)	5.0 (4.0-6.0)	5.2 (4.2-6.9)	5.0 (4.0-6.5)	5.2 (4.0-7.0)	5.0 (4.0-6.2)	5.7 (4.2-7.5)	5.0 (4.2-6.7)	5.4 (4.1-7.1)
Arrest duration, median (IQR), min	20 (15-27)	12 (10-15)	12 (9-16)	18 (15-22)	20 (16-25)	22 (18-26)	22 (19-27)	26 (22-30)	25 (21-30)	27 (21-32)	28 (23-35)	31 (24-38)	31 (24-40)
Missing arrest duration	1295 (24.7)	141 (24.6)	140 (23.2)	154 (20.5)	145 (28.0)	140 (20.4)	116 (28.8)	100 (22.2)	92 (28.3)	70 (24.7)	45 (31.0)	80 (26.0)	72 (34.6)
Prehospital care													
Intraosseous access only	707 (13.5)	35 (6.1)	43 (7.1)	111 (14.8)	69 (13.3)	102 (14.9)	60 (14.9)	74 (16.4)	55 (16.9)	42 (14.8)	36 (24.8)	39 (12.7)	41 (19.7)
Advanced airway	5186 (98.7)	561 (97.7)	571 (94.7)	747 (99.5)	509 (98.3)	685 (100.0)	401 (99.5)	447 (99.3)	324 (99.7)	281 (99.3)	145 (100.0)	307 (99.7)	207 (99.5)
Hospital care													
TTM use	3052 (58.1)	574 (48.8)		751 (59.2)		685 (63.0)		450 (58.0)		283 (66.1)			308 (59.7)
Stent	570 (10.9)	104 (18.1)	64 (10.6)	84 (11.2)	45 (8.7)	73 (10.7)	31 (7.7)	49 (10.9)	14 (4.3)	44 (15.5)	8 (5.5)	38 (12.3)	16 (7.7)
Implantable defibrillator	605 (11.5)	195 (34.0)	89 (14.8)	105 (14.0)	31 (6.0)	76 (11.1)	16 (4.0)	38 (8.4)	10 (3.1)	14 (4.9)	2 (1.4)	25 (8.1)	4 (1.9)
Outcomes													
Survived to hospital discharge	2177 (41.4)	469 (81.7)	471 (78.1)	362 (48.2)	211 (40.7)	201 (29.3)	107 (26.6)	104 (23.1)	69 (21.2)	69 (24.4)	22 (15.2)	74 (24.0)	18 (8.7)
CPC 1-2 neurologic status	1889 (36.0)	441 (76.8)	436 (72.3)	299 (39.8)	177 (34.2)	169 (24.7)	89 (22.1)	85 (18.9)	48 (14.8)	53 (18.7)	17 (11.7)	58 (18.8)	17 (8.2)

Abbreviations: AED, automatic external defibrillator; CPC, Cerebral Performance Category; CPR, cardiopulmonary resuscitation; EMS, emergency medical services; TTM, targeted temperature management.

^a^ Data are presented as number (percentage) of patients unless otherwise indicated. Data are complete, with the exception of arrest duration, with number (percentage) missing.

^b^ Restricted to those who had arrested before EMS arrival.

We observed a dose-dependent association between increasing epinephrine dose and decreasing neurologically favorable survival. Neurologically favorable survival was 74.5% (877 of 1177) for those receiving 0 mg, 37.5% (476 of 1269) for more than 0 to 1 mg, 23.7% (258 of 1088) for more than 1 to 2 mg, 17.2% (133 of 775) for more than 2 to 3 mg, 16.4% (70 of 428) for more than 3 to 4 mg, and 14.5% (75 of 516) for more than 4 mg. In the logistic regression model, higher doses of prehospital epinephrine were associated with decreasing odds of neurologically favorable survival (odds ratio [OR], 0.46; 95% CI, 0.42-0.50 for each additional milligram of epinephrine) and survival to hospital discharge (OR, 0.47; 95% CI, 0.43-0.51 for each additional milligram of epinephrine) after adjusting for Utstein covariates. Overall, neurologically favorable survival did not differ according to TTM status (36.2% for TTM vs 35.6% for no TTM).

**Figure 2.**
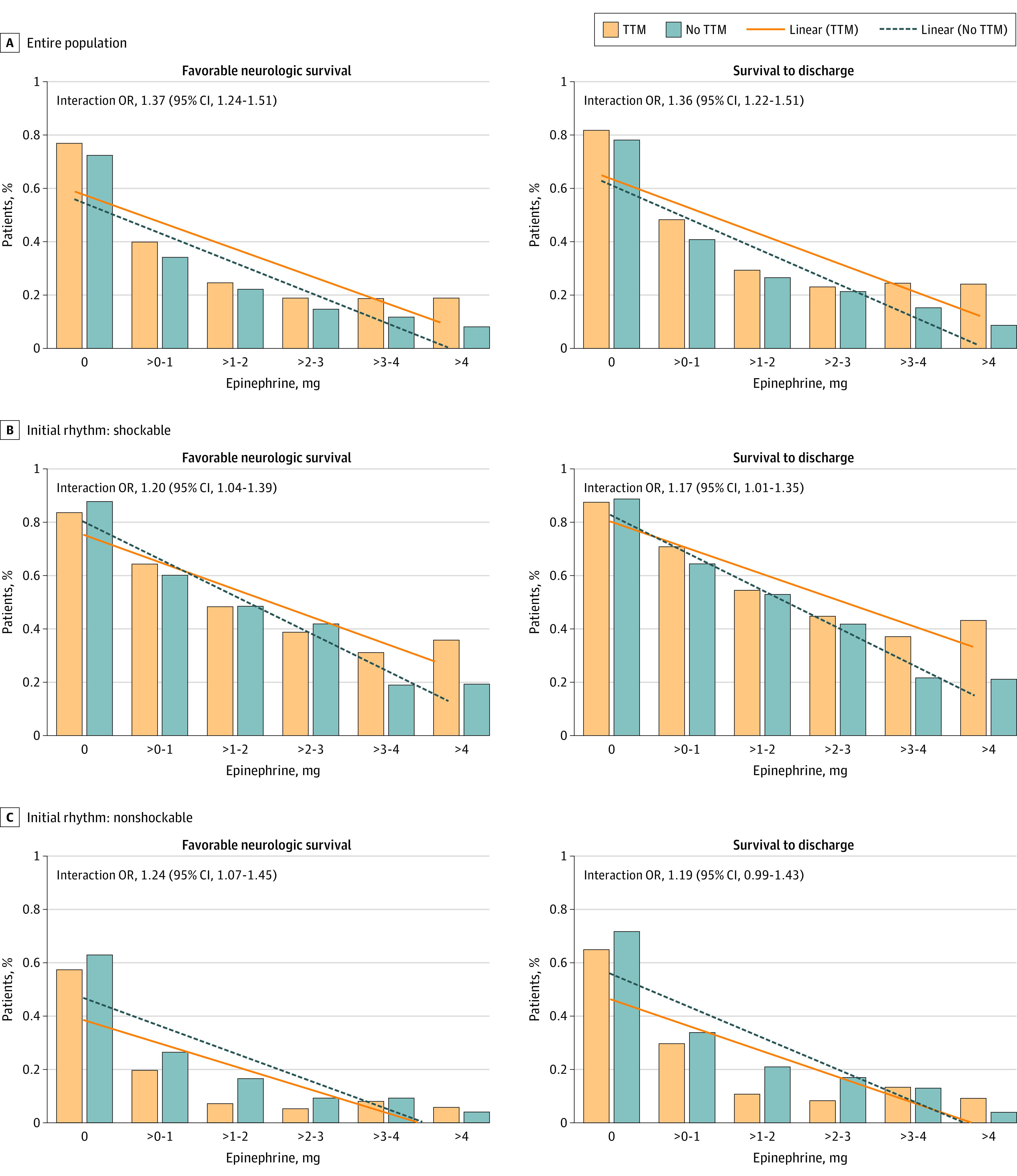
Targeted Temperature Management (TTM), Epinephrine Dose, and Clinical Outcome After Out-of-Hospital Cardiac Arrest Functional survival is defined as Cerebral Performance Categories 1 to 2. The interaction odds ratio (OR) is the OR of a good outcome associated with TTM for each additional 1-mg increase in epinephrine, modeling epinephrine × TTM interaction using a continuous epinephrine dose variable. The multivariable mixed-effect model adjusted for Utstein covariates, including age, sex, witness status (witnessed by emergency medical services, witnessed by bystander, and unwitnessed), bystander cardiopulmonary resuscitation, automatic external defibrillator application, initial rhythm (shockable vs nonshockable), and the interval from receipt of 9-1-1 call to initial responder scene arrival, using hospital as a random effect.

When epinephrine dose was modeled as a continuous variable, the association between outcome and increasing epinephrine dose was modified by TTM (Figure 2). After adjustment for Utstein covariates, TTM compared with no TTM was associated with a relative stepwise improvement in odds of neurologically favorable survival (interaction OR, 1.36; 95% CI, 1.22-1.51, for each additional milligram of epinephrine) and survival (interaction OR, 1.37; 95% CI, 1.24-1.51 for each additional milligram of epinephrine). The significant interaction indicates that the slope of outcome vs epinephrine dose is different according to TTM status, with the decrease in both clinical survival outcomes being less steep among those who received TTM. A significant interaction was also observed when the analysis was stratified according to initial rhythm among shockable OHCA and nonshockable OHCA (shockable interaction OR, 1.20; 95% CI, 1.04-1.39 and nonshockable interaction OR, 1.24; 95% CI, 1.07-1.45) (Figure 2).

**Figure 3.**
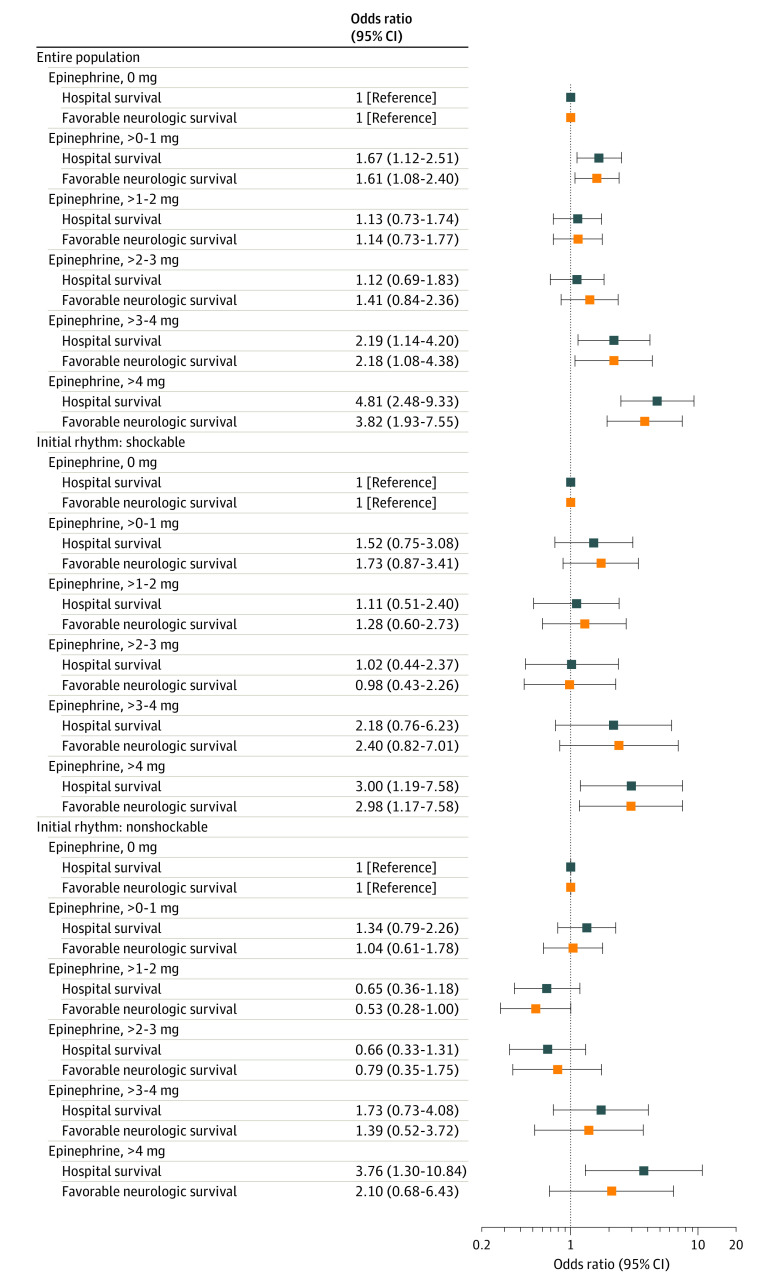
Association of Targeted Temperature Management (TTM) and Clinical Outcome According to Epinephrine Dose After Out-of-Hospital Cardiac Arrest The odds ratios represent the association of TTM (vs no TTM) with clinical outcome for each epinephrine dose modeling epinephrine × TTM interaction term using an independent categorical epinephrine variable at 1-mg increments. Neurologically favorable survival defined as Cerebral Performance Categories 1 to 2. The multivariable mixed-effect model adjusted for Utstein covariates, including age, sex, witness status (witnessed by emergency medical services, witnessed by bystander, and unwitnessed), bystander cardiopulmonary resuscitation, automatic external defibrillator application, initial rhythm (shockable vs nonshockable), and the interval from receipt of 9-1-1 call to initial responder scene arrival, using hospital as a random effect.

**Figure 4.**
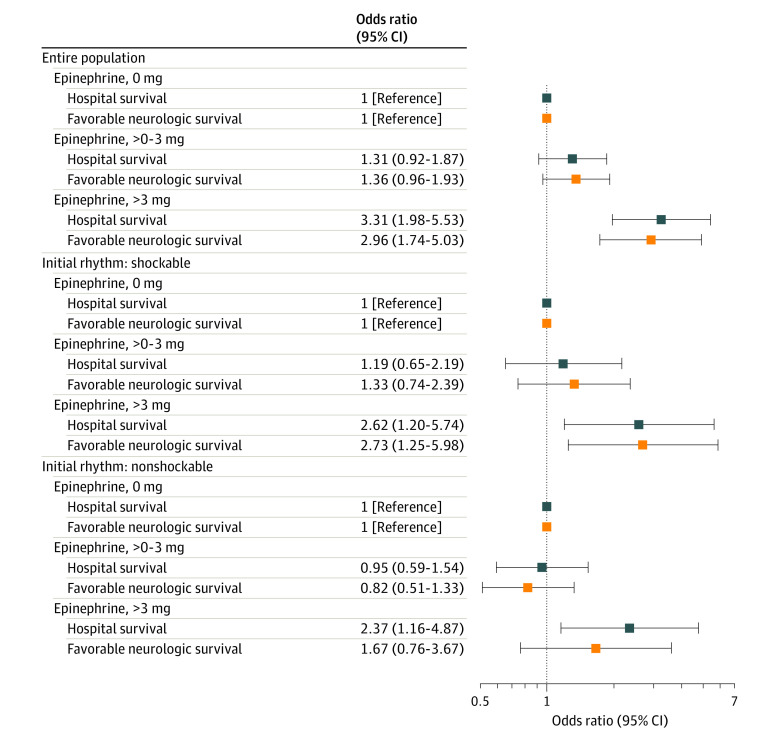
Association of Targeted Temperature Management (TTM) and Clinical Outcome According to Epinephrine Dose After Out-of-Hospital Cardiac Arrest The odds ratios represent the association of TTM (vs no TTM) with clinical outcome for each epinephrine dose group modeling epinephrine × TTM interaction term using an independent categorical epinephrine grouped variable. Neurologically favorable survival is defined as Cerebral Performance Categories 1 to 2. The multivariable mixed-effect model adjusted for Utstein covariates, including age, sex, witness status (witnessed by emergency medical services, witnessed by bystander, and unwitnessed), bystander cardiopulmonary resuscitation, automatic external defibrillator application, initial rhythm (shockable vs nonshockable), and the interval from receipt of 9-1-1 call to initial responder scene arrival, using hospital as a random effect.

When epinephrine dose was modeled as an independent categorical variable, TTM was associated with a benefit when the dose exceeded 2 mg for both favorable neurologic survival and survival to discharge (Figure 3). In the post hoc evaluation in which epinephrine dose was modeled as 0, more than 0 to 3 mg, and more than 3 mg, TTM was associated with neurologically favorable survival benefit at higher doses of epinephrine but not lower doses of epinephrine (Figure 4). Results were comparable when the different epinephrine exposure models were additionally adjusted for duration of arrest (eTable 2 in the Supplement).

## Discussion

### Summary

In this cohort study of patients resuscitated from OHCA and eligible for TTM, we evaluated whether TTM modified the adverse association between increasing epinephrine dose and decreasing likelihood of neurologically favorable survival. We observed a significant interaction that indicated the relative favorable survival benefit associated with TTM increased with higher doses of epinephrine. There was little evidence of clinical outcome benefit associated with TTM among those with no or low-dose epinephrine; in contrast, TTM was associated with 3-fold greater odds of neurologically favorable survival among those receiving more than 3 mg of epinephrine during field resuscitation. The dose-dependent interaction between epinephrine and TTM was consistent among those who presented with shockable and nonshockable rhythms. The findings suggest that dose of prehospital epinephrine may identify patients who may especially benefit from TTM.

### Epinephrine Primary Outcome

In the current study, we observed that increasing dose of epinephrine was associated with decreasing likelihood of neurologically favorable survival, an association previously described.^8,22^ Multiple explanations may account for the adverse dose association. Increasing epinephrine may be a surrogate for refractory patient physiologic features, indicating that increasing epinephrine dose is simply a marker of disease severity. Alternatively, epinephrine may contribute directly to pathology because the balance of benefit and risk changes with increasing dose. Some evidence suggests that the cerebral macrovascular and microvascular effects of epinephrine are dose dependent, with increasing dose reducing cerebral perfusion and brain tissue oxygenation.^6,7^ In addition to the total dose of epinephrine administered during resuscitation, the timing of epinephrine dosing has been associated with outcomes, highlighting the complexity of the association between epinephrine and outcomes.^23^

### TTM Outcomes: The Concept of Interaction

Given this understanding of epinephrine, we evaluated whether TTM might have differential effects based on epinephrine dose. Epinephrine-related decreases in cerebral blood flow and brain tissue oxygenation may be offset or matched by lower cerebral oxygen consumption in patients receiving TTM.^6,7^ In addition, TTM may provide neuroprotection by limiting the complex pathophysiologic mechanism of ischemia-reperfusion injury.^14^ This injury is presumably characterized by a spectrum of severity that might respond differentially to TTM. Patients with more severe ischemia-reperfusion injury might benefit from TTM more than patients with less severe injury. We hypothesized that patients with more severe injury characterized by higher doses of epinephrine might benefit relatively more from TTM. The results from the current investigation support this concept of a differential effect of TTM that depends on the epinephrine dose.

### Alternative Explanations

There are other potential explanations for our results. As noted, higher doses of epinephrine may simply be a marker of ischemic burden, such that the interaction really reflects a more generic association between increasing beneficial effects of TTM among those with greater disease severity. Increasing epinephrine dose was associated with longer estimated duration of resuscitation, although adjustment for arrest duration did not substantially change the association among epinephrine dose, TTM, and outcome (eTable 2 in the Supplement). Alternatively, TTM requires dedicated and extended critical care that often precludes early withdrawal of support or comfort care, efforts that may be especially beneficial among those with the most serious insult. Thus, the relative neurologically favorable survival and overall survival benefit of TTM observed with higher doses of epinephrine may identify the differential effect of comprehensive critical care among this sickest cohort, as opposed to a specific benefit related to mitigating epinephrine brain injury.

### Limitations

This study has some limitations. The study is observational; thus, the results provide for association but not causality. Moreover, confounding may contribute to the observed associations. For example, the study lacks information about prearrest clinical comorbidities, whether the patient experienced rearrest, or the decision and timing related to goals of care, characteristics that may be associated with the dose of epinephrine, the use of TTM, and the likelihood of survival. The study was not able to assess the dose (duration and temperature) or mode of TTM, which may potentially have a therapeutic role, although studies^8,22^ of the duration and degree of TTM have yet to identify differential effects. Guidelines indicate that patients who remain comatose after resuscitation are eligible for TTM. The current study excluded those who were determined to be awake or conscious in the ED but was not able to apply a more refined coma scale, such as the Glasgow Coma Score, to classify coma. The study occurred in a relatively high-performing system where bystander CPR is common, EMS response is relatively quick, and the median dose of epinephrine in the current cohort was 2 mg, all characteristics that may have implications for generalizability. These limitations should be considered in the context of the study’s strengths: the investigation evaluated an important clinical question using a relatively large cohort with robust prehospital and hospital covariate and outcome measures.

## Conclusions

Among patients resuscitated from OHCA, the adverse association between increasing epinephrine and neurologically favorable survival was modified by TTM. The relative benefit of TTM increased with higher doses of epinephrine, suggesting that TTM may have a differential effect, depending on intra-arrest treatment, in this case the total dose of epinephrine. Whether such an understanding can better direct therapy to improve survival is uncertain and requires additional investigation.

## Notes

Supplement. **eTable 1.** Patient Characteristics Stratified by TTM **eTable 2.** Association of Targeted Temperature Management (TTM) and Clinical Outcome According to Epinephrine Dose Following Out-of-Hospital Cardiac Arrest
