# Supplementary material for: Analysis of Epinephrine Dose, Targeted Temperature Management, and Neurologic and Survival Outcomes Among Adults With Out-of-Hospital Cardiac Arrest
Source: JAMA Netw Open. 2022 Aug 11;5(8):e2226191. doi: 10.1001/jamanetworkopen.2022.26191 (PMC9372792; doi:10.1001/jamanetworkopen.2022.26191)
Supplement: Supplement. — eTable 1. Patient Characteristics Stratified by TTM eTable 2. Association of Targeted Temperature Management (TTM) and Clinical Outcome According to Epinephrine Dose Following Out-of-Hospital Cardiac Arrest [file jamanetwopen-e2226191-s001.pdf]

Supplementary Online Content

Yang BY, Bulger N, Chocron R, et al. Analysis of epinephrine dose, targeted temperature management, and neurologic and survival outcomes among adults with out-of-hospital cardiac arrest. *JAMA Netw Open*. 2022;5(8):e2226191. doi:10.1001/jamanetworkopen.2022.26191

eTable 1. Patient Characteristics Stratified by TTM

eTable 2. Association of Targeted Temperature Management (TTM) and Clinical Outcome According to Epinephrine Dose Following Out-of-Hospital Cardiac Arrest

This supplementary material has been provided by the authors to give readers additional information about their work.

© 2022 Yang BY et al. *JAMA Network Open.*

eTable 1. Patient Characteristics Stratified by TTM

Epinephrine Dose	TTM Yes	TTM No
Characteristics n	3051	2202
Age, years (median [IQR])	62 [51, 73]	64 [52, 76]
Female sex, n (%)	928 (30.4)	865 (39.3)
Male sex, n (%)	2123 (69.6)	1337 (60.7)
Cardiac Etiology Arrest, n (%)	2314 (75.8)	1166 (53.0)
Arrest before EMS arrival, n (%)	2788 (91.4)	1755 (79.7)
Public Location (%)	891 (29.2)	465 (21.1)
Witnessed Arrest[a], n (%)	1721 (61.7)	1070 (61.0)
Bystander CPR[a], n (%)	1898 (68.1)	1195 (68.1)
Shockable Initial Rhythm, n (%)	1506 (49.4)	566 (25.7)
PAD Application (%)	245 (8.0)	140 (6.4)
Call to EMS Arrival, min (median [IQR])	5.0 [4.0, 6.1]	5.2 [4.0, 6.8]
Arrest duration, min (median [IQR])	20.6 [15.4, 26.6]	20.2 [13.8, 27.0]
Missing arrest duration, n(%)	685 (22.5)	610 (27.7)
Prehospital Care		
Intraosseous Access Only, n (%)	403 (13.2)	304 (13.8)
Advanced Airway, n (%)	3029 (99.3)	2157 (98.0)
Hospital Care		
TTM Use, n (%)	3051 (58.0)	
Stent, n (%)	392 (12.8)	178 (8.1)
Implantable Defibrillator, n (%)	453 (14.8)	152 (6.9)
Outcomes		
ROSC at end of EMS Care, n (%)	2927 (95.9)	2092 (95.0)
Survived to Hospital Discharge, n (%)	1279 (41.9)	898 (40.8)
CPC 1-2 Neurological Status, n (%)	1105 (36.2)	784 (35.6)

[a] Restricted to those who had arrested before EMS arrival.
Data elements are complete, with the exception of Duration of Arrest, with n (%) missing as indicated.
Abbreviations: TTM = Targeted Temperature Management, CPR = cardiopulmonary resuscitation, AED = Automatic External Defibrillator, ROSC = Return of Spontaneous Circulation, CPC = Cerebral Performance Category.

© 2022 Yang BY et al. *JAMA Network Open.*

eTable 2. Association of Targeted Temperature Management (TTM) and Clinical Outcome According to Epinephrine Dose Following Out-of-Hospital Cardiac Arrest

		Adjusted Odds Ratio (95% CI)		
**Model**	**Epinephrine**	**Overall Population**	**Shockable Population**	**Nonshockable Population**
**Survival to discharge**				
Continuous Interaction		1.47 (1.30-1.66, p<0.001)	1.31 (1.09-1.57, p=0.003)	1.27 (1.06-1.53, p=0.012)
Categorical Epi dose (1 mg increments)	0 mg	1	1	1
	>0-1 mg	1.89 (1.18-3.04, p=0.008)	1.86 (0.79-4.37, p=0.156)	1.55 (0.85-2.81, p=0.151)
	>1-2 mg	1.18 (0.70-1.98, p=0.530)	0.97 (0.37-2.57, p=0.951)	0.71 (0.36-1.43, p=0.338)
	>2-3 mg	1.31 (0.73-2.33, p=0.361)	1.06 (0.38-2.94, p=0.913)	0.86 (0.39-1.91, p=0.714)
	>3-4 mg	2.47 (1.12-5.41, p=0.024)	1.86 (0.55-6.32, p=0.319)	1.99 (0.69-5.72, p=0.203)
	>4 mg	7.56 (3.16-18.10, p<0.001)	4.63 (1.43-14.97, p=0.010)	7.10 (1.39-36.20, p=0.018)
Categorical Epi dose as 0, >0-3, >3 mg	0 mg	1	1	1
	>0-3 mg	1.44 (0.95-2.19, p=0.085)	1.28 (0.60-2.72, p=0.522)	1.11 (0.65-1.91, p=0.702)
	> 3 mg	4.27 (2.26-8.07, p<0.001)	3.19 (1.21-8.42, p=0.019)	3.05 (1.22-7.62, p=0.017)
**Survival with CPC 1-2**				
Continuous Interaction		1.50 (1.31-1.71, p<0.001)	1.26 (1.05-1.51, p=0.012)	1.26 (1.00-1.59, p=0.050)
Categorical Epi dose in 1 mg increments	0 mg	1	1	1
	>0-1 mg	1.82 (1.14-2.92, p=0.012)	2.05 (0.91-4.62, p=0.082)	1.14 (0.61-2.12, p=0.674)
	>1-2 mg	1.30 (0.77-2.21, p=0.329)	1.35 (0.53-3.44, p=0.531)	0.56 (0.26-1.20, p=0.138)
	>2-3 mg	1.71 (0.92-3.19, p=0.091)	1.03 (0.38-2.79, p=0.950)	1.11 (0.42-2.91, p=0.832)
	>3-4 mg	2.46 (1.04-5.82, p=0.040)	2.03 (0.59-7.05, p=0.264)	1.61 (0.45-5.74, p=0.460)
	>4 mg	5.57 (2.30-13.49, p<0.001)	4.18 (1.31-13.33, p=0.016)	3.48 (0.63-19.15, p=0.152)
Categorical Epi dose as 0, >0-3, >3 mg	0 mg	1	1	1
	>0-3 mg	1.55 (1.03-2.33, p=0.036)	1.49 (0.73-3.03, p=0.276)	0.92 (0.53-1.61, p=0.781)
	> 3 mg	3.78 (1.94-7.35, p<0.001)	3.11 (1.20-8.08, p=0.020)	2.16 (0.75-6.19, p=0.154)

The ORs represent the association of TTM (versus no TTM) with clinical outcome for each epinephrine dose group modeling epinephrine X TTM interaction term using 3 separate models. The first models epinephrine as a continuous variables, the second models epinephrine as independent categorical epinephrine grouped in 1 mg increments, and the third models epinephrine as independent categorical epinephrine grouped by 0 mg, >0-3 mg, and >3 mg. Neurologically favorable survival defined as Cerebral Performance Categories (CPC) 1-2. The multivariable mixed-effect model adjusted for Utstein covariates, including age, sex, witness status (witnessed by EMS, witnessed by bystander, and unwitnessed), bystander CPR, automatic external defibrillator (AED) application, initial rhythm (shockable versus non-shockable), and the interval from receipt of 9-1-1 call to initial responder scene arrival, using hospital as a random effect.

TTM = Targeted Temperature Management, CPR = cardiopulmonary resuscitation, AED = Automatic External Defibrillator, ROSC = Return of Spontaneous Circulation, CPC = Cerebral Performance Category.

© 2022 Yang BY et al. *JAMA Network Open.*
